# Supplementary material for: Serine/Threonine Protein Phosphatases 1 and 2A in Lung Endothelial Barrier Regulation
Source: Biomedicines. 2023 Jun 5;11(6):1638. doi: 10.3390/biomedicines11061638 (PMC10296329; doi:10.3390/biomedicines11061638)
Supplement: Supplementary file 1 [file biomedicines-11-01638-s001.zip › biomedicines-2406392-supplementary.pdf]

Table S1. Shown are specific primers of PP2A 'B' isoforms expression profile in HLMVECs obtained by PCR

Gene	Gene Accession # *Homo sapiens*	Name	Forward Primer / Reverse primer	Product size (bp)
PPP1R1A	NM_014225.5	PP2A Aα	TCATGGGTCTCTCTCCCATC<br>TAGTCATGCGGTGCAGGTAG	456
PPP2R1B	NM_002716.4	PP2A Aβ	CAGAAAGCCATGGGTCCTAA<br>CAGCAGCGGCATATACTCAA	480
PPP2R2A	NM_002717.3	PP2A Bα	GTTTGACAGTGTGCCATTCG<br>CCACTGACCCCAAACTGACT	484
PPP2R2B	NM_004576.2	PP2A Bβ	GGAACTGGAGCTGGAGTGAG<br>GGTTCATGGCTCTGGAATGT	489
PPP2R2C	NM_020416.3	PP2A Bγ	CAGCCCTAGAGGCAGTTGAC<br>GCGTCTCAGTTCTCCTGGAC	470
PPP2R2D	NM_018461.3	PP2A Bδ	CGGAGGAAAGACGAGATCAG<br>AGGAAGGCTCGGACTGAAAT	431
PPP2R3A	NM_002718.4	PP2A B′′α	AGCAGAGGGAGCCTATCACA<br>GGGGCTTTCCAATAGAGAGG	464
PPP2R3C	NM_017917.2	PP2A B′′γ	GCAAGCAATTTTTCACAGCA<br>TTAGGGCAGAAGGAGCAGAA	429
PPP2R4	NM_021131.4	PP2A B′ (activator)	CGCCAGATTCTTCAGAGGAG<br>ACTGTGGCCACCAAGTTTTC	448
PPP2R5A	NM_006243.3	PP2A B′α	CATTGCCACTGAAAGCAGAA<br>TGGCAAACATAATTGGCAGA	411
PPP2R5B	NM_006244.3	PP2A B′β	GAGCGGGCTCTGTATTTCTG<br>GTCCTCAGAAGCCACTCTGG	494
PPP2R5C	NM_002719.3	PP2A B′γ	AGTTGGCCAAATGTGTCTCC<br>GAGCCTCGTCCTTCACTGTC	450
PPP2R5D	NM_006245.3	PP2A B′δ	GCTCCCTACTGGCTGTCTTG<br>CAGGCATGGGGTAAGAAGAA	492
PPP2R5E	NM_006246.3	PP2A B′ε	AGAGGTCGCAAAGTTCCTCA<br>TCTTGGCTTTCCAAAAATCG	415

PP2A: Protein Phosphatase 2A; HLMVECs: Human Lung Microvascular Endothelial Cells; PCR: Polymerase Chain Reaction; bp: base pair.
